# The heterogeneous and dynamic immune-tumor interface in breast cancer

**DOI:** 10.3389/fonc.2026.1906555

**Published:** 2026-08-07

**Authors:** Constantin N. Baxevanis, Maria Goulielmaki, Angelos D. Gritzapis, Ourania E. Tsitsilonis

**Affiliations:** 1 Cancer Immunology and Immunotherapy Center, St. Savas Cancer Hospital, Athens, Greece; 2 Flow Cytometry Unit, Department of Biology, National and Kapodistrian University of Athens, Athens, Greece

**Keywords:** breast cancer, cancer progression, immune checkpoint blockade, immune escape, immune exhaustion, suppression, tertiary lymphoid structures, tumor microenvironment

## Abstract

Breast cancer (BCa) progression is driven by ongoing interactions between evolving tumor cells and the host immune system. Antitumor immunity is not the same across all BCa subtypes and depends on the quality, localization, and functional state of immune cells within the tumor microenvironment (TME). Highly immunogenic subtypes, such as triple-negative and HER2-positive (+) BCa, generally show stronger immune infiltration and inflammatory signaling, whereas estrogen receptor–positive tumors more often display immune-desert or immune-excluded phenotypes with limited immune cell infiltration and effector activity. Effective immune surveillance requires not only the presence of immune cells, but also their suitable spatial localization and functional capacity. Cytotoxic CD8+ T cells mediate tumor control when they infiltrate tumor areas, whereas stromal-related limitations or functional exhaustion reduces their efficacy. This dysfunction is reinforced by regulatory populations, including FoxP3+ regulatory T cells and CD163+ tumor-associated macrophages, which establish suppressive networks that inhibit effector responses. Within this framework, T-cell immunity is organized into distinct functional states, including stem-like progenitor exhausted T cells, which maintain their immune potential, and effector T cells responsible for cytotoxicity, which support local surveillance. Tertiary lymphoid structures further enhance coordinated immune activation by serving as intratumoral sites of antigen presentation and lymphocyte priming. Immune escape arises through T-cell exhaustion, impaired antigen presentation, tumor-intrinsic signaling changes, and stromal barriers that restrict infiltration. Collectively, these mechanisms generate a regulated, but often tumor-supporting immune environment. BCa progression therefore, reflects a balance between immune activation and suppression, governed by spatial organization and cellular stratification within the TME.

## 1 Introduction

Breast cancer (BCa) is best understood as a biologically and immunologically heterogeneous disease in which tumor progression is shaped not only by intrinsic genetic alterations but also by dynamic interactions with the host immune system (1, 2). BCa evolves through a continuously changing equilibrium between malignant cells and immune surveillance mechanisms, with the intensity and quality of this interaction varying across molecular subtypes (2, 3). Consequently, immune pressure acts as a context-dependent factor that shapes cancer evolution by eliminating some tumor cell clones while selecting others. This variability is particularly evident in immunogenic subtypes such as triple-negative and HER2+ BCa, which frequently exhibit higher levels of tumor infiltrating lymphocytes (TILs), enhanced interferon (IFN) signaling, and upregulated inflammatory gene expression (4–6). These features are partly driven by greater genomic instability and increased tumor mutational burden, which promote neoantigen generation, facilitate immune-mediated tumor recognition and guide therapeutic treatments (7, 8). In contrast, estrogen receptor–positive tumors tend to exhibit more immunologically “quiet” (i.e., inactive) phenotypes. These include immune-desert states, where immune cells are largely absent, and immune-excluded states, where immune cells are present but trapped in stromal regions without effective tumor infiltration (9–11). These differences are not simply descriptive but reflect a substantially distinct tumor–immune crosstalk, regulated by both tumor biology and the tumor microenvironment (TME) (9, 10, 12).

One of the most important advances in BCa research has been the recognition that immune infiltration alone is insufficient to predict clinical outcomes. Tumors with comparable levels of immune cell presence can exhibit markedly different behaviors depending on whether these immune cells are functionally active, spatially localized near tumor nests, or suppressed by regulatory mechanisms (9, 13, 14). In BCa, this has moved the field beyond the simplistic “immune-hot versus immune-cold” classification toward a more detailed framework that considers combining immune cell functionality, spatial organization and cellular state (activation versus exhaustion) (9, 11, 12).

Tumor-intrinsic signaling pathways play a central role in shaping the breast tumor immune microenvironment. Activation of WNT/β-catenin signaling has been implicated in immune exclusion, partly through the suppression of dendritic cell recruitment and impaired T-cell priming (15, 16). Similarly, aberrant PI3K/AKT pathway activation, frequent in hormone receptor–positive BCa, and MYC-driven transcriptional programs contribute to immune evasion by disrupting antigen presentation, promoting metabolic competition, and altering cytokine signaling (17, 18). These mechanisms collectively reduce tumor immunogenicity and limit effective anti-tumor immune responses. In parallel, the breast tumor stroma provides an additional layer of immune regulation. Extracellular matrix remodeling, increased tissue density, and aberrant vascularization act as physical and biochemical barriers that restrict immune cell trafficking and infiltration into tumor nests (19, 20). These structural features interact with tumor-intrinsic pathways to reinforce immune resistance and shape distinct immune phenotypes across BCa subtypes. In the following sections, we examine how the composition, spatial organization, and functional states of immune cells shape antitumor immunity in BCa. We first discuss the biological and clinical significance of TILs and tertiary lymphoid structures (TLS), followed by the regulatory networks that drive immune suppression and escape. Finally, we consider the implications of these mechanisms for immunotherapy and emerging strategies aimed at restoring effective immune surveillance.

## 2 Spatial organization of tumor-infiltrating lymphocytes

TILs have long been recognized as one of the most informative immune-related biomarkers in BCa, but their biological and subsequently clinical interpretation has become increasingly complex over time. Initial studies primarily considered TILs as a quantitative measure, demonstrating that higher stromal TILs density is associated with improved prognosis and better response rates to chemotherapy, especially in highly immunogenic subtypes such as triple-negative and HER2+ BCa (4, 5, 21). This established their role as a clinically useful prognostic and predictive marker. However, this early perspective overlooked that TILs vary not only in number but also in function, location within the tumor, and activation state (being active, dysfunctional, or exhausted).

More recent research has significantly modified this view by emphasizing that the predictive value of TILs depends not only on their abundance but also on their spatial organization, functional state, and interaction with both tumor and stromal compartments (22–24). In this broader view, immune infiltration is not uniform but, instead, it is a highly organized process shaped by tissue architecture and local signals within the TME. It is important that immune cells must not only be present but also correctly positioned to effectively exert their antitumor activity (23, 25). Spatial immune organization has therefore become a central determinant of immune effectiveness in BCa. CD8+ cytotoxic T cells that infiltrate directly into tumor epithelial nests are strongly associated with tumor control and improved survival outcomes (26–28). In contrast, CD8+ cytotoxic T cells that remain restricted to stromal regions, sometimes even at high densities, often display reduced cytotoxicity and limited interaction with autologous malignant cells (27, 28). This spatial separation highlights a critical concept in tumor immunology, namely that immune exclusion is not just the absence of immune cells, but rather a failure of immune cells to access tumor epithelial compartments and interact with tumor cells (22, 25, 29). Further, this spatial restriction is actively shaped by suppressive immune populations which are often positioned at the interface between stromal and epithelial regions, where they can effectively block immune cell infiltration. They suppress antitumor immunity through multiple mechanisms, including secretion of inhibitory cytokines, expression of immune checkpoint ligands, and competition for key metabolic resources such as glucose and tryptophan, which are essential for T cell activation and function (9, 29–31). Advances in spatial transcriptomics, multiplex imaging, and single-cell profiling have further reinforced the importance of immune cell positioning over simple immune cell densities (22, 32, 33). These technologies showed that tumors with similar TILs densities can still have very different immune landscapes, depending on whether immune cells are arranged in immune-promoting rather than in suppressive stromal compartments. In this context, BCa should be considered as a spatially organized immune environment, whereby the effectiveness of antitumor immunity depends on the precise localization of immune cells relative to tumor cell areas, the presence of physical barriers, and the distribution of suppressive immune networks that shape activation and functional competence.

## 3 The role of tertiary lymphoid structures in systemic immune interactions

TLS represent one of the most important spatially organized immune features, reflecting a level of immune organization that goes beyond simple infiltration of immune cells into the tumor tissue. These lymphoid aggregates resemble secondary lymphoid organs such as the lymph nodes, yet they form *de novo* within chronically inflamed tumor environments. TLS development is generally associated with sustained antigen exposure and ongoing immune activity, and they function as localized centers for antigen presentation, lymphocyte activation, and adaptive immune regulation (32, 34). In BCa, the presence of TLS has been consistently linked with improved prognosis, reduced recurrence risk, and enhanced responsiveness to both chemotherapy and immunotherapy, suggesting that they reflect a more competent and self-sustaining antitumor immune state (32, 34–36).

From a structural perspective, TLS are highly organized clusters of immune cells. They typically contain B-cell follicles with germinal center-like activity, T cell-rich zones, networks of dendritic cells, and high endothelial venules that facilitate the continuous recruitment of circulating lymphocytes (37, 38). This architecture allows for efficient local priming and differentiation of immune cells directly within the TME, bypassing the need for antigen transport to distant lymph nodes. As a result, TLS can function as autonomous immune-processing units that sustain ongoing immune responses over time. They also support affinity maturation of B cells and clonal expansion of tumor-specific T cells, thereby strengthening both the humoral and cellular arms of antitumor immunity (32, 34, 38). In BCa, TLS are increasingly recognized as robust spatial biomarkers of antitumor immunity, with emerging evidence suggesting they may provide more reliable prognostic and predictive information than programmed cell-death protein (PD)-1/PD-ligand (L)1 expression or conventional TILs quantification (35, 36, 39). In both early and advanced BCa, particularly in triple-negative and HER2+ subtypes, TLS presence is associated with an inflamed TME characterized by coordinated B- and T-cell activation and improved clinical outcomes (4, 5, 21, 35, 39).

Within the context of immune checkpoint blockade (ICB) targeting the PD-1/PD-L1 axis, TLS appear to play a key role in stratifying responders from non-responders, likely by supporting local antigen presentation, sustaining T-cell priming, and reinforcing intratumoral immune activation (32, 34, 38). One possible explanation is that tumors enriched with TLS are more likely to possess pre-existing immune memory which can be further enhanced by ICB, whereas TLS-poor tumors often exhibit immune exclusion and reduced responsiveness to therapy. However, TLS biology in BCa is highly heterogeneous and context-dependent. Their formation, maturation state, and spatial organization within the TME can differentially influence tumor progression, immune activation, and clinical outcomes. While mature, germinal center–like TLS are generally associated with favorable prognosis, immature or poorly organized structures may reflect incomplete immune activation (34, 38, 40–42). This complexity shows that TLS could be useful biomarkers for treatment decision-making and may be targets for future immune-based therapies in BCa. However, at present, their clinical interpretation remains challenging.

## 4 Regulatory feedback mechanisms and immune suppression

Immune regulation under normal physiological conditions is controlled by complex and coordinated feedback networks that are essential for maintaining immune homeostasis. These systems are designed to prevent excessive immune activation and preserve self-tolerance. However, within the TME, these regulatory pathways are often reprogrammed to support immune escape and tumor persistence (1, 9). Instead of just blocking immune responses, they assist in the formation of a tumor-supporting environment that actively influences how immune cells behave as cancer progresses. Regulatory T cells (Tregs), particularly those characterized by FoxP3 expression, represent one of the most important mediators of immune suppression in BCa. Tregs inhibit cytotoxic CD8+ T-cell activity through multiple complementary mechanisms. They secrete immunosuppressive cytokines such as interleukin (IL)-10 and transforming growth factor (TGF)-β, which directly decrease effector T-cell activation and proliferation. In addition, they consume available IL-2, creating metabolic competition that limits effector T-cell expansion. Tregs also express inhibitory checkpoint ligands that further suppress immune activation and contribute to local immune exhaustion (1, 43). These mechanisms allow Tregs to exert a broad control over antitumor immunity. Tumor-associated macrophages (TAMs), particularly CD163+M2-like subsets, further amplify this suppressive environment. TAMs promote extracellular matrix remodeling, support angiogenesis, and secrete cytokines that reinforce immune suppression and tissue repair programs rather than inflammatory responses (44–46). In this way, they contribute not only to the inhibition of antitumor immunity but also, due to structural changes within the TME, restrict immune infiltration (45, 47). Importantly, Tregs and TAMs do not function independently but form spatially organized networks that often colocalize with tumor cell clusters or tumor compartments where effector T cells are present. This spatial localization creates areas of immune suppression that effectively dampen cytotoxic activity even in the presence of immune infiltration (1, 48). The result is a dynamic and constantly evolving equilibrium between activation and inhibition (49, 50). In many breast tumors, however, this equilibrium progressively shifts toward immunosuppression. As tumor evolution progresses, suppressive networks expand in both number and intensity, thereby controlling local immune responses and ultimately enabling tumor growth (51). This imbalance is continuously shaped by interactions between the tumor and the immune system. For instance, when immune activation increases, whether through natural immune surveillance or therapeutic treatment, suppressive mechanisms often become more pronounced in parallel, thus restricting effective tumor control. This adaptive feedback loop is a major contributor for the development of resistance to immunotherapy and represents one of the key barriers for achieving durable immune-mediated tumor regression (52).

## 5 Therapeutic implications and reinvigoration of the antitumor immunity

Immunotherapy in BCa is more likely to be effective when a pre-existing antitumor memory immune response is already present in the TME, rather than depending on the initiation of a completely new one. In this context, immune checkpoint inhibitors, such as PD-1 and PD-L1 blockers, primarily exert their effects by targeting stem-like progenitor exhausted T cells, which retain their proliferative capacity and the potential to differentiate into functional effector populations once inhibitory signaling is abrogated (53–55). These progenitor-like cells generate a reinvigorated antitumor immunity, and their presence appears to be decisive of whether a tumor can respond meaningfully to therapy. However, clinical outcomes vary widely because this responsive immune landscape is not homogenously present across BCa subtypes. Tumors that lack stem-like T-cell populations or are characterized by strong immune exclusion phenotypes tend to show limited or no responses to ICB, even when immune cells are detected in the surrounding stroma (56). This has modified the therapeutic view toward the idea that the effectiveness of immunotherapy depends not only on immune activation, but also on how easily immune cells can access the tumor and the type of cells present within the TME. As a result, combination strategies have become a major focus of current clinical research. Conventional treatments such as chemotherapy and radiotherapy are now recognized not only for their direct cytotoxic effects but also for their capacity to enhance antitumor immunity. These therapies can induce immunogenic cell death, leading to the release of tumor-associated antigens, activation of dendritic cells, and subsequent priming of T-cell responses (57). In this way, they can make “cold” tumors inflamed and recognizable by the immune system.

Targeted therapies also contribute to immune modulation. In HER2+ BCa, for example, HER2-directed monoclonal antibodies can enhance antibody-dependent cellular cytotoxicity and in this way, promote immune cell recruitment, effectively linking targeted oncogenic inhibition with immune activation. Cellular immunotherapies (including chimeric antigen receptor (CAR) T-cell therapies and T-cell receptor (TCR)-T cell therapies), as well as T-cell bispecific antibody therapies show especially strong potential (58, 59). Similar immune-modulatory effects have been observed with other pathway-targeted agents that alter tumor signaling and antigen presentation (60, 61). Overall, effective therapeutic strategies in BCa require more than simply stimulating immune activity. They need to overcome barriers that block immune cells from entering tumors, persisting within the TME, and staying functional over time. For this, therapeutic modalities should work in a way to overcome spatial immune exclusion, reverse T-cell exhaustion and disrupt suppressive networks formed by Tregs and TAMs. Only by addressing both activation and accessibility, durable immune-mediated tumor control can be achieved.

## 6 Immune escape mechanisms and T- cell exhaustion

Immune escape in BCa is driven by coordinated changes in tumor cells and immune regulation, rather than by a single key pathway. A central determinant of immune failure is CD8+ T-cell exhaustion, a progressive differentiation state driven by chronic antigen exposure and sustained involvement of inhibitory immune checkpoint pathways (55, 62). In this context, persistent signaling through receptors such as PD-1, cytotoxic T lymphocyte antigen (CTLA)-4, T-cell immunoglobulin and mucin domain (TIM)-3, lymphocyte activation gene (LAG)-3, and T-cell immunoreceptor with Ig and ITIM domains (TIGIT) becomes a major feature of dysfunctional T cells, actively inhibiting their cytotoxic activity (63–66). Importantly, checkpoint receptor expression is not stable, as exhausted T cells display co-expressed inhibitory receptor profiles that reflect distinct stages of dysfunction and responsiveness to immunotherapy (62, 65, 67). Transcription factors such as TOX sustain exhaustion and maintain T cells into durable dysfunctional states characterized by reduced cytokine production, impaired proliferative capacity, metabolic reprogramming, and diminished cytotoxicity (62, 67). Despite this dysfunction, exhausted T cells are not eliminated but persist within the TME, where high checkpoint receptor expression maintains a reversible but dampened functional state. This biology provides the mechanistic basis for the clinical efficacy of ICB, which primarily reinvigorates exhausted T cells by disrupting PD-1/PD-L1–mediated inhibitory signaling and restoring effector differentiation potential in a subset of progenitor-like exhausted cells.

Although ICB has significantly advanced cancer immunotherapy, a substantial proportion of initially responding patients ultimately develop resistance and experience disease progression. Mechanisms of tumor-intrinsic resistance to ICB include multiple molecular and immunological alterations that impair effective anti-tumor immunity (**Table 1**).

**Table 1:** Mechanisms of tumor-intrinsic resistance to immune checkpoint blockade.

Alteration	Molecular mechanism	Effect on tumor microenvironment	Impact on response to ICB	Key refs
IFN-γ signaling defects (IFN-γR1/2, JAK1/2, IRF1)	Loss of IFN-γ responsiveness → impaired STAT1 signaling, reduced expression of MHC-I, and CXCL9/10	Non-inflamed TME with poor T-cell recruitment and activation	Primary and acquired resistance due to lack of immune engagement	(68, 69)
Loss of neoantigen-coding mutations	Immunoediting eliminates highly immunogenic mutations	Reduced antigenicity and T-cell priming	Poor response due to absence of targetable epitopes	(70)
Loss of neoantigen-expressing tumor clones	Clonal selection eliminates immunogenic subclones under immune pressure	Decreased clonal diversity and immune visibility	Adaptive resistance and tumor escape	(70, 71)
Irreversible T-cell exhaustion (restricted TCR-Vβ)	Chronic antigen exposure → epigenetically stabilized exhausted state (PD-1^+^ high, TOX^+^)	Dysfunctional T cells with limited proliferative capacity	Limited reinvigoration by ICβ	(72)
Expression of multiple immune checkpoints (PD-L1, CTLA-4, TIM-3, LAG-3, TIGIT)	Redundant inhibitory signaling pathways	Highly suppressive TME with layered inhibition	Reduced efficacy of single-agent ICB; need for combinations	(64, 66, 73)
WNT/β-catenin activation	Suppression of CCL4 → failure to recruit BATF3^+^ dendritic cells	Immune-desert phenotype with absent T-cell infiltration	Strong primary resistance to ICB	(16, 74)
CASPASE-8 loss	Impaired extrinsic apoptosis (Fas/TRAIL pathways) → resistance to cytotoxic cell killing	Tumor survives despite T-cell infiltration	Functional resistance to immune-mediated killing	(16, 74, 75)
Antigen processing machinery (APM) defects	Alterations in TAP1/2, tapasin, proteasome subunits → impaired peptide loading	Reduced antigen presentation on MHC-I	Immune evasion despite T-cell activation	(68, 69, 76)
β2-microglobulin (β2M) mutations	Loss of MHC-I surface expression due to instability of the HLA complex	Complete lack of CD8^+^ T-cell recognition	Strong resistance to ICB	(77)
Loss (or downregulation) of HLA	Reduced diversity or expression of HLA alleles	Narrowed antigen presentation repertoire	Immune escape under selective pressure	(78)

IFN-γ, interferon-γ; JAK 1/2, Janus kinase 1/2; IRF1, interferon regulatory factor 1; STAT1, signal transducer and activator of transcription 1; MHC, major histocompatibility complex; PD-L1, programmed cell-death protein-ligand 1; CXCL9/10, C-X-C motif chemokine ligand 9/10; TME, tumor microenvironment; TCR-Vβ, T-cell receptor variableβ; TOX, thymocyte selection-associated high mobility group box protein; CTLA-4, cytotoxic T lymphocyte antigen-4; TIM-3, T-cell immunoglobulin and mucin domain-3; LAG-3, lymphocyte activation gene-3; TIGIT, T-cell immunoreceptor with immunoglobulin and ITIM domains; ICB, immune checkpoint blockade; CCL4, C-C motif chemokine ligand 4; BATF3, basic leucine zipper ATF-like transcription factor 3; TRAIL, TNF-related apoptosis-inducing ligand; TAP, transporter associated with antigen processing; HLA, human leucocyte antigen.

**Figure 1:**
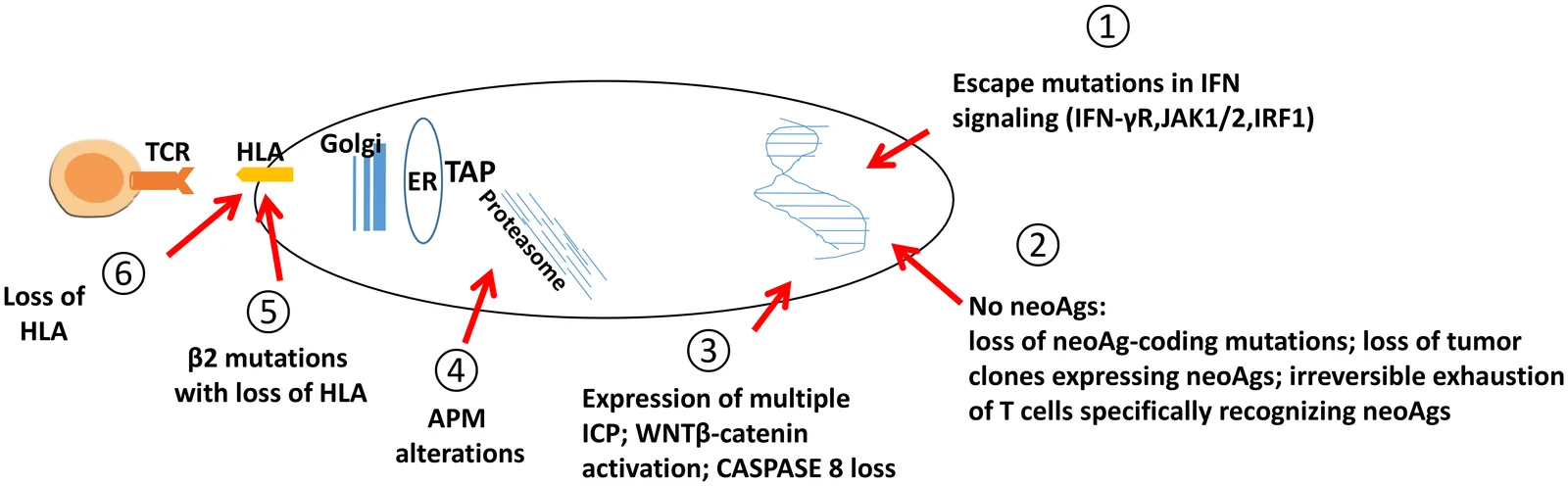
Intrinsic mechanisms of acquired immune resistance to immune checkpoint blockade (ICB). Tumor-intrinsic resistance to ICB arises from multiple genetic and immunological alterations that impair anti-tumor immunity. (1) Defects in interferon signaling (interferon-γ receptor, Janus kinase 1/2, interferon regulatory factor 1) reduce IFN-γ responsiveness, lowering MHC I expression, antigen presentation, and chemokine production, resulting in a non-inflamed TME. (2) Tumors also evade immunity through loss of neoantigens via immunoediting, clonal loss, or T-cell exhaustion, diminishing T-cell recognition. (3) Upregulation of multiple immune checkpoints creates redundant inhibitory pathways that limit checkpoint blockade efficacy. Activation of WNT/β-catenin signaling excludes dendritic cells and T-cell priming, promoting immune exclusion. Loss of Caspase 8 reduces susceptibility to T-cell–mediated apoptosis. (4-6) Additional resistance mechanisms include defects in antigen-processing machinery, β2-microglobulin mutations causing loss of HLA class I expression, and HLA loss of heterozygosity or downregulation, all of which impair antigen presentation and facilitate immune escape. IFN-γR, interferon-γ receptor; JAK 1/2, Janus kinase 1/2; IRF1, interferon regulatory factor 1; TME, tumor microenvironment; TAP, transporter associated with antigen processing; ER, endoplasmatic reticulum; HLA, human leucocyte antigen.

Escape mutations in IFN signaling components such as IFN-γ receptor, Janus kinase 1/2, and IFN regulatory factor 1, reduce tumor responsiveness to IFN-γ, leading to decreased major histocompatibility complex (MHC) class I expression, impaired antigen processing, reduced chemokine production, and failure to establish an inflamed TME, ultimately resulting in immune ignorance and resistance to ICB (68, 69). Tumors may also evade immunity through loss of neoantigens, either by immunoediting that eliminates highly immunogenic mutations, by selective loss of tumor clones expressing neoantigens, or by inducing irreversible exhaustion of T-cell receptors due to chronic antigen exposure, all of which reduce T-cell recognition and function, and promote a “cold” TME (70–72). Additionally, tumors often upregulate multiple immune checkpoints, creating redundant inhibitory pathways and compensatory mechanisms that limit the effectiveness of single-agent checkpoint blockade (64, 66, 73). Activation of the WNT/β-catenin pathway further contributes to immune exclusion by preventing dendritic cell recruitment and T-cell priming, resulting in a non–T-cell-inflamed phenotype associated with primary resistance (16, 74). Loss of CASPASE-8 confers resistance to apoptosis and reduces susceptibility to T-cell-mediated cytotoxicity, even in the presence of TILs (16, 74, 75). Alterations in antigen processing machinery components impair peptide processing and presentation on MHC class I molecules, leading to defective CD8^+^ T-cell recognition (68, 69, 76). Mutations in β2-microglobulin result in complete loss of human leucocyte antigen (HLA) class I surface expression and abolish antigen presentation to CD8^+^ T cells, conferring strong resistance to immune-mediated killing (77). Finally, loss of HLA through genetic loss of heterozygosity or transcriptional downregulation reduces antigen presentation diversity and enables tumors to escape immune recognition under selective pressure (78) (see also **Figure 1**).

All these mechanisms create a multilayered network of immune resistance in which checkpoint receptor–driven T-cell exhaustion acts as a key point, integrating chronic antigen exposure, tumor-derived suppression, and microenvironmental limitations. Functional overlap across these pathways explains the robustness of immune escape in BCa and highlights why durable antitumor immunity is rarely achieved through single-agent interventions. Instead, effective immune reactivation requires coordinated disruption of multiple suppressive pathways in order to restore antigen presentation and reprogramme dysfunctional T-cell states. Within this framework, ICB represents a foundational but, nevertheless, incomplete strategy, as it primarily targets inhibitory receptor signaling without fully resolving tumor-intrinsic defects or the broader immunosuppressive contexture of the TME. Accordingly, next-generation therapeutic approaches increasingly focus on combination strategies that integrate ICB with agents that reduce suppression and increase tumor immunogenicity establishing inflamed TMEs. Together, these efforts aim not only to reinvigorate exhausted CD8+ T cells but also to re-establish a permissive immune environment capable of sustaining long-lasting antitumor responses in BCa.

## 7 Conclusion

BCa immunobiology is driven by a dynamic system in which local and systemic immune responses are closely interconnected. Across all BCa subtypes, antitumor immunity is not determined simply by immune cell quantity but by the spatial organization and functional capacity of immune populations within the TME. TLS provide an important structure for immune activation inside tumors, linking local antigen presentation with broader systemic immune responses and reflecting ongoing immune activity. Within this framework, T-cell immunity plays an essential role in maintaining antitumor surveillance and effective antitumor responses. However, the effectiveness of this system is continuously shaped, and often limited, by dominant immunosuppressive networks, including Tregs and CD163+TAMs, which establish spatially defined barriers to immune function and reinforce tumor tolerance. Tumor progression and therapeutic resistance therefore develop from a dynamic equilibrium between immune activation and suppression, further combined by exhaustion pathways, antigen presentation defects, and stromal exclusion mechanisms. Together, these findings imply that BCa is a spatially organized malignancy whose clinical outcomes are determined not solely by individual immune components, but by the coordinated function of a complex, dynamic, and adaptable tumor immune microenvironment.
